# Supplementary material for: Molecular mechanisms of immune evasion by host protein glycosylation of a bacterial immunogen used in nucleic acid vaccines[image]
Source: J Biol Chem. 2026 Apr 2;302(5):111423. doi: 10.1016/j.jbc.2026.111423 (PMC13188097; doi:10.1016/j.jbc.2026.111423)

Supplementary Figures

Supplementary Figure 1: A representative spectrum of N52 glycan site demonstrates the rich fragmentation generated from the stepped high-energy collisional dissociation (sHCD), which provides functional fragments both of the peptide backbone and of the glycan itself in a single MS2, which are used by the search engine pGlyco3 to identify glycopeptides.

Supplementary Figure 2: Ag85B is microheterogeneously glycosylated when expressed in human Expi293 cells. Site-specific microheterogeneity of **(a)** N52, **(b)** N224, **(c)** N234, and **(d)** N280 using glycomics-informed glycopeptidomics. Relative spectral counts were normalized to the most abundant glycoforms present at each site. Complex structures are represented in dotted orange, hybrid structures are represented in checkered purple, and high-mannose structures are represented in triangle light green.

Supplementary Figure 3: Hierarchical clustering of molecular dynamics simulations for the **(a)** nonglycosylated and **(b)** glycosylated Ag85B in ten representative conformations.

Supplementary Figure 1

293-F Ag85B N52 –H3N4F1 composition MS2

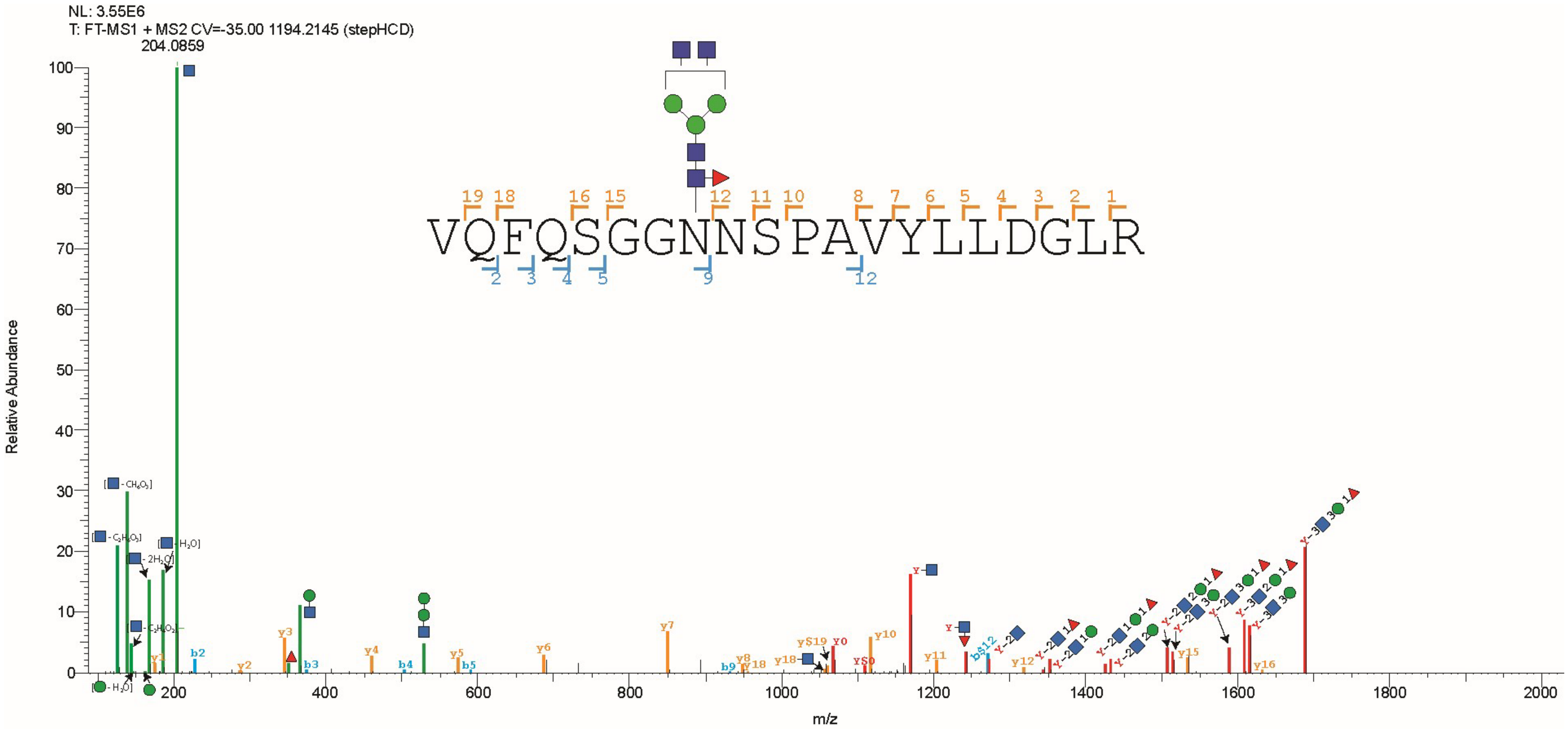

Supplementary Figure 2

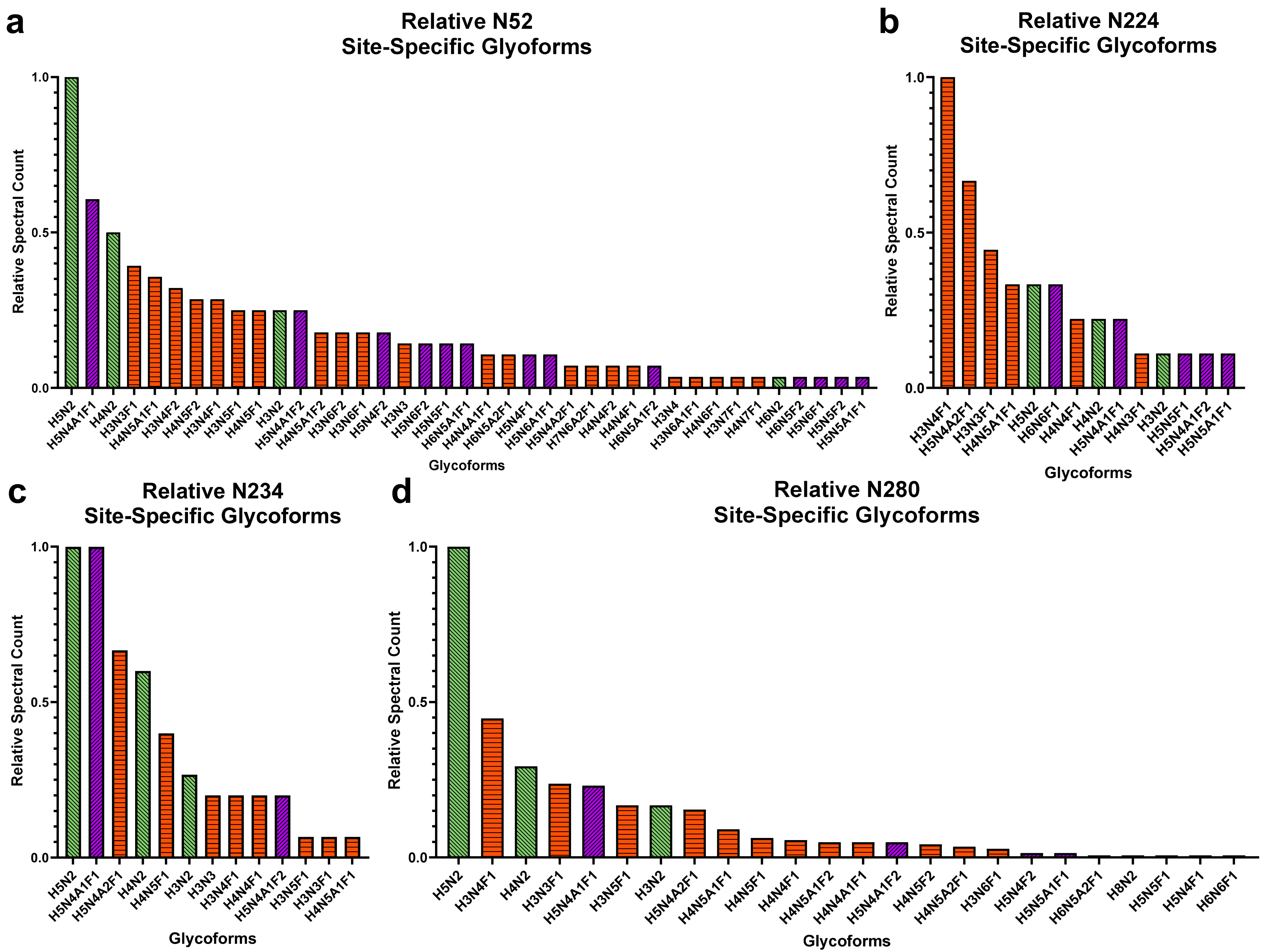

Supplementary Figure 3

a **Nonglycosylated**

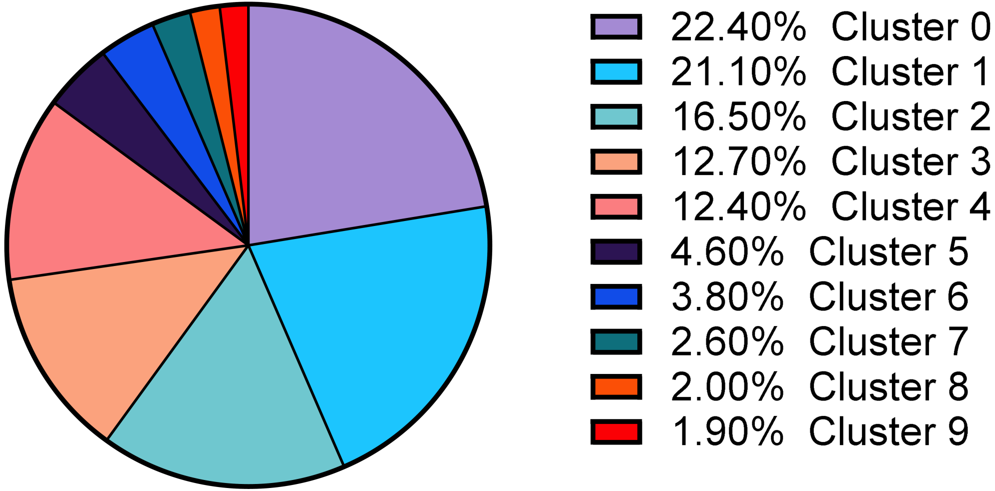

b **Glycosylated**

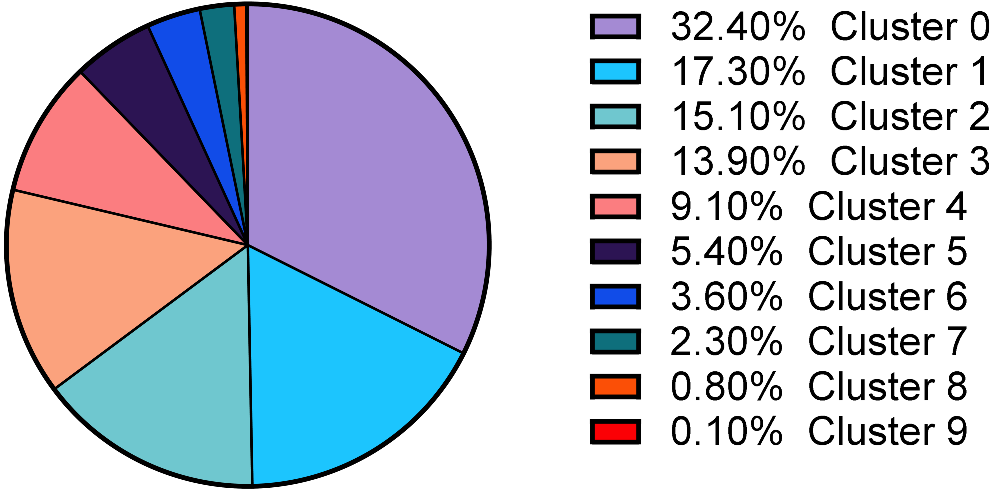

Supplement: Supplement Figures [file mmc1.pdf]
